# An evaluation of South Africa’s public–private partnership for the localisation of vaccine research, manufacture and distribution

**DOI:** 10.1186/s12961-018-0303-3

**Published:** 2018-03-27

**Authors:** David R. Walwyn, Adolph T. Nkolele

**Affiliations:** Department of Engineering and Technology Management, University of Pretoria, Private Bag X20, Hatfield, Pretoria, 0028 South Africa

**Keywords:** vaccines, public–private partnership, cost-benefit analysis, value-for-money

## Abstract

**Background:**

Public–private partnerships (PPPs), widely used as a means of leveraging the skills, expertise and resources of the private sector to mutual advantage, were similarly adopted by South Africa to support public sector delivery. This study has evaluated one such partnership, namely the Biovac Institute, which was established in 2003 to cover vaccine research and development, manufacturing, and supply. The initiative was highly unusual given that it attempted to combine all three aspects in a single PPP.

**Methods:**

The research has followed a concurrent mixed methods approach. In the quantitative study, data for prices and product volumes were extracted from secondary data sources and used to calculate the economic cost and value-for-money of the PPP. Simultaneously, a qualitative study was undertaken in which a number of key stakeholders were interviewed using a semi-structured questionnaire on their perceptions of the PPP’s value.

**Results:**

The institute earns a premium on the procurement cost of a broad range of vaccines required by the South African National Department of Health for its immunisation programme, the net value of which was US$85.7 million over the period 2010 to 2014. These funds were used to finance the institute’s operations, including vaccine research, distribution and quality control. Capital expenditure to support the establishment of facilities for laboratory testing, packaging and labelling, filling, formulation and, finally, active pharmaceutical ingredient manufacture, approximately US$40 million in total, had to be secured through loans and grants. According to the respondents in the qualitative survey, the principal benefit of the PPP has been the uninterrupted supply of vaccines and the ability to respond quickly to vaccine shortages. The main disadvantages appear to have been a slow and ineffectual establishment of a vaccine manufacturing centre and, initially, a limited ability to negotiate highly competitive vaccine prices.

**Conclusions:**

Overall, it is concluded that a positive value-for-money has been achieved and the institute has been of significant public benefit. Relationships of this nature can be used to achieve public health goals, but need to be realistic about timeframes, costs and the limitations of relational governance in ensuring that complex programmatic outcomes are achieved. It is recommended that a more incremental approach, with clearer contractual goals, penalties and incentives, is adopted in attempting initiatives aimed at the localisation of manufacturing technology by leveraging public procurement.

## Background

Vaccines are lauded as one of the most successful public health interventions, providing universal prophylaxis at a fraction of the cost that would otherwise be incurred following the widespread outbreak of an infectious disease. It is estimated that every dollar invested in immunisation delivers a return of US$16 in terms of savings in public healthcare costs and increased economic productivity [1, 2]. As a result, WHO, through the activities of the Global Vaccine Action Plan, is working to raise levels of vaccination coverage to at least 90% by 2020, especially for the critical diseases of diphtheria-tetanus-pertussis, *Haemophilus influenzae* type b, hepatitis B, measles and polio, all of which are effectively and affordably controlled by the present vaccines and share the common characteristic of being highly contagious and having severe disease outcomes, especially for children.

South Africa already has an extensive vaccination programme, known as the Expanded Program on Immunisation (EPI), which forms part of the broader health strategy as adopted by the National Department of Health (NDoH) and is intended to “*prevent death and reduce suffering from infections that can be prevented by the immunisation of children and women*” ([3], p. 4). Implementation of the EPI requires the procurement of approximately 46 million vaccine doses annually, at a cost of roughly 1.5 billion Rand per annum (2015 values). Prior to 2003, vaccine procurement was an internal function of the NDoH; the department issued tenders on behalf of the provinces and secured the necessary supply from successful bidders. However, since 2004, vaccine procurement and distribution has been undertaken by a public–private partnership (PPP), known as the Biovac Institute (BI).

The formation of a PPP to provide an essential function of public health services raises a number of interesting and important questions about the role of the public sector, and the extent to which this can be satisfactorily shared or outsourced to the private sector. Given that a critical responsibility for the public sector is to provide and protect public goods, and that the objective of the private sector is primarily the pursuit of private gain, there exists a clear, structural conflict of interest within such an arrangement. The very notion of a partnership, in which one party is bound by a social contract and the other by a private contract, must therefore be carefully framed by an acknowledgement and understanding of this conflict [4, 5]. Although it is accepted that PPPs adopt a mutual commitment to a well-defined outcome (such as the establishment of local vaccine manufacturing infrastructure) through a structured cooperation, which goes beyond the “*principal-agent dynamic of a contractual relationship*” and within which the risks, costs and benefits are shared equally [6], in practice, PPPs may lead to incomplete or poor implementation [7], limited risk transfer to the private partner [8], and a breakdown of trust at all levels [4, 9].

Indeed, the expectation that PPPs will lead to improved value-for-money (VfM), reduced life cycle costs, higher levels of innovation, and risk mitigation may be misplaced [10]; PPPs that do not adopt specific measures for strong governance, tight performance management and strict transparency, despite the apparent contradiction of these measures to the notion of a partnership, may at best simply fail to deliver public value but at worst result in unbridled rent seeking by the private partner [11]. Moreover, PPPs have been described as ‘incomplete contracts’, implying that they are more likely to encounter a set of unanticipated events that will negatively influence the partnership and may lead to loss of control by the public entity [12].

In this article, we describe the results of a VfM, or cost-benefit analysis, of the BI-PPP. There are already in the literature several hundred publications on PPPs, covering a range of sectors and partnership types. However, very few deal with manufacturing and, as far as the authors are aware, there are no reports of PPPs covering vaccine procurement, distribution and supply. Other gaps in the present literature include the limited use of longitudinal studies or research approaches other than case studies, the limited application of surveys for data acquisition, and a narrow scope of PPP evaluation, which tends to be restricted to cost-benefit analysis and ignores other factors such as social return on investment and employee satisfaction [13]. The latter are important since PPPs are also social constructs in which two parties from different sectors, cultures and perspectives agree on the partial or complete privatisation of a specific service previously supplied by the public sector. The impact of this decision on workers and the broader public is infrequently studied and reported [14].

This study has attempted to address at least some of these gaps in the literature. It has followed a concurrent mixed methods approach with a qualitative arm adopting a survey approach to assess stakeholder perceptions of BI’s contribution to public health. Further, it has followed a longitudinal approach to the collection of quantitative data and it deals with a previously unreported application of PPPs, namely the use of the PPP structure to support the transfer and localisation of vaccine manufacturing technology. The use of public procurement as a means of stimulating innovation, employment and economic growth is particularly relevant to developing countries, given the opportunity that demand-side measures offer in this respect along with the general difficulty in translating this opportunity into real outcomes [15–17].

In the first section, the general theoretical framework for PPPs is described followed by an overview of South Africa’s immunisation programme and the formation of BI. Details of the research questions and methodology are then presented, leading to the results and, finally, a detailed discussion of the implications thereof. The article concludes with an overall comment on the value of the PPP and how similar projects within the general area of public health could be addressed in the future.

### Overview of PPPs

#### General theory

Although there are common elements to all PPPs, including a mutually agreed definition of the intended outcome, such as the establishment of some form of public infrastructure achieved through mutual commitment and dedication to the partnership, PPPs cover a broad range of intersectoral initiatives in which the two partners share varying levels of risk, benefit, resources and responsibilities within a contractual relationship, which can vary considerably from simple contracting to a long-term shared accountability [13, 18].

A suitable framework for understanding this diversity has been described by Brinkerhoff and Brinkerhoff [6], who define the two dimensions of mutuality and organisational identity through which PPPs can be classified. The former refers to the extent to which the partners share control, decision-making and responsibility whereas the latter covers the unique competencies, capabilities, markets and comparative advantages. An ideal PPP is considered as a partnership in which mutuality is high but organisational identities are retained throughout the project. This form of PPP can be easily separated from conventional contracting (high organisational identity, low mutuality), extension of competence (low organisational identity, low mutuality) and eventual absorption (low organisational identity, high mutuality).

Other frameworks use a typology based on a separation of responsibilities between the various stages of a partnership, namely the design and conceptualisation, the construction, operations and maintenance, and final ownership [18, 19]. In the case of the World Bank classifications, which relate mainly to infrastructure projects, the broad categories are supply and management contracts, turnkey projects, affermage/leasing (build-lease-transfer), concessions, and private ownership/private finance initiatives [18]. Within the latter two categories are a group of options including build-own-operate, build-own-operate-transfer and build-transfer-operate.

A key success factor for PPPs is the effective allocation of risk, which includes the initial identification of project risk factors followed by the allocation of these factors to that party which is best able to manage them [20, 21]. Neither tasks are straightforward given the complexity of the political, economic, social and technical environments, as well as the long time frames within which large PPP projects operate. Indeed, such risks are typically under-identified or allocated with the result that projects fail to meet their targets, including those of scope, time and budget [20].

Another aspect of the PPP relationship, indeed of many contractual arrangements, is that it is typically characterised by an information asymmetry, in which at least one party is in certain areas or respects more knowledgeable than the other, thereby creating an imbalance in power relations and/or benefits. In PPPs, such asymmetry can be the source of conflict and eventual breakdown of the partnership, as may be expected. In order to avoid such tension, it is essential that the two parties have frequent and transparent interaction that allows the sharing of information and helps to build trust within the venture.

Information exchange between the two parties is only one aspect of a whole set of social mechanisms that are critical in partnerships shaped by the principles of relational governance, where the latter emphasises the role of trust, flexibility, solidarity and actor networks as a means of preventing the exploitation of the PPP [22]. Relational governance is a construct defined in contrast to contractual governance, where the latter is enforced through explicit contracts that provide the legal and institutional framework and define, in as much detail as possible, the rights, duties and responsibilities of each party [22].

In practice, most PPPs combine both governance arrangements in a complementary fashion, thereby achieving a more efficient outcome than if either approach were to be pursued on its own [23]. Although contractual governance may appear to offer the most protection from default by either party, highly detailed and comprehensive contracts are expensive to prepare, inflexible and difficult to monitor. Moreover, such contracts can never cover every eventuality and, as a consequence, may be ineffective instruments to enforce the rights of each party [22]. As noted below, the PPP of this study was initially shaped by a detailed set of shareholder contracts; however, as time progressed, relational governance became more important, perhaps even dominant. Such dynamics are not unusual in PPPs and have been reported in prior studies [22].

### PPPs in South Africa

South Africa followed a global trend in the popularity of PPPs by establishing a more formal PPP structure within the National Treasury in 1999 [24]. Although there were PPPs prior to this date, these arrangements did not follow a standardised process or receive formal recognition as PPPs within the treasury. Following the launch of the PPP unit in 1999, the National Treasury (of South Africa) developed a standardised procedure for such an entity, which it defined as a “*contract between a government institution and a private party, where the private party performs an institutional function and/or uses state property in terms of output specifications; substantial project risk (financial, technical, operational) is transferred to the private party; and the private party benefits through unitary payments from government budgets and/or user fees*” [25].

The documentation further proceeded to define the important qualifying factors for PPPs, which include risk transfer to the private sector (does the PPP result in the transfer of financial or project risk which may be incurred including the risk of time overruns, revenue projections and operational costs?), affordability (is the project within existing budget constraints?) and VfM (will the PPP be less costly than the state-owned alternative?). All PPPs were required to follow an approval process in which these questions were formally answered as part of the rationale for the partnership.

The treatment of risk is somewhat contradictory considering that the nature of a PPP is such that both parties, but particularly the state, are subject to additional risk as a result of asymmetry and incomplete contracting. It could be argued that PPPs are a means by which both parties accept an open-ended relationship in which risks are shared rather than transferred. This aspect implies that, inevitably, expectations of the state (the public party) may never be realised; risk remains an ongoing and visceral property of a PPP that can never be entirely transferred or avoided.

### Formation of the BI

As mentioned above, BI was established in 2003 through a strategic equity partnership with the Biovac Consortium (Pty) Ltd., where the latter was a private company comprised of a number of shareholders, namely Biovac Holdings (62.5%), Heber Biotec (15%), VaxIntel (15%) and the Disability Employment Concern Trust (7.5%). The Biovac Consortium held a controlling share in BI (52.5%), with the remainder being held by the NDoH. The latter shareholding was subsequently transferred to the Department of Science and Technology.

The partnership was initiated after the NDoH acknowledged the deterioration in South Africa’s vaccine production assets and hence the need to access external competency in this critical area. It was apparent from the earliest stages of the PPP that its objective was not to address issues of vaccine supply chain management, which was already being outsourced to the suppliers of vaccines within the immunisation programme and for which there were no immediate concerns given that shortages or supply failures to the various clinics and depots within the country had not occurred. Neither were there issues of tendering and contracting; the NDoH was already, very successfully, implementing other procurement processes for the purchase of essential medicines, including the antiretroviral programme. Instead, the PPP was motivated by the need to maintain security of supply through local manufacturing, a tradition which had its origins in earlier projects and had resulted in the manufacturing facilities for a number of vaccines including polio and Bacillus Calmette–Guérin (BCG), where the latter is used to prevent tuberculosis. This central rationale was later captured in the core agreements of the PPP, and specifically the Shareholders Agreement, as shown in Table 1.

**Table 1:** List of Biovac Institute public–private partnership objectives

Category	Objective
Vaccine production (Capacity)	Ensure a domestic capacity in vaccine production that will enable the South African health authorities to respond to disease outbreak emergencies
Vaccine production (Quality)	Establish an economically viable vaccine producer applying the principles of current good manufacturing practice
Vaccine production (Skills)	Develop and retain local vaccine production-related skills and ensure the continued development of biotechnology and related skills
Research and development	Establish a strong research and development capability focused on the development of locally relevant vaccines
Markets (Exports)	Create a competitive platform from which a domestic producer of human vaccines and related medical biotechnology products can compete with other markets
Markets (General)	Ensure that any development in this sector in South Africa opens access to other markets as potential customers
Black economic empowerment (BEE)	Promote BEE and the identification of a BEE partner to participate through a shareholding in the Biovac Institute

Source: Naidoo [44]

The financing of these objectives, and the overall model for the PPP, was to set aside a portion of the procurement costs, henceforth referred to as the price premium, for the funding of the capital investment and new product development programmes, as required by the objectives. The latter was covered by two separate facilities, namely the State Vaccine Institute, which was manufacturing an intravenous BCG and developing a rabies vaccine, and the South African Vaccine Producers, which was developing a combination vaccine for diphtheria-tetanus-pertussis. Both facilities had been poorly assessed by a recent WHO report that had noted that the products were redundant (e.g. intravenous BCG had been replaced by intradermal BCG) and the facilities themselves were unlikely to pass a formal WHO inspection [26]. Similarly, the Transaction Adviser for the PPP declared in the Option Analysis that “*SAVP* [South African Vaccine Producers] *has no commercial value and the SVI* [State Vaccine Institute] *has no commercial value from a going concern perspective … in the event of liquidation, its value may be between R3 million and R5 million*” [27].

The original agreements, as signed in 2004, consisted of a Supply Agreement, the Shareholders Agreement, the Subscription Agreement and the Strategic Equity Partner Undertakings. The agreements initially covered the period 2004 to 2010, but were subsequently renewed to the end of 2016. The Supply Agreement dealt with the NDoH outsourcing of procurement, central level storage, and distribution of vaccines to nine provincial vaccine storage depots [28]. In addition, a separate distribution agreement was signed between BI and a provincial Department of Health to distribute vaccines from the depots to the clinics (at a 6% premium) [29]. It is noted that these agreements were central to the governance framework of the PPP, and as such the partnership can be defined, at least initially, as being dominated by contractual governance. However, the circumstances of BI and the somewhat unrealistic targets of the Strategic Equity Partner Undertakings resulted in the governance shifting to a relational basis, leading to some flexibility in the application of the various agreements.

As defined by the Supply Agreement, BI charged the NDoH both the purchase cost of vaccines and the price premium where the latter varied between 10% and 20%, and covered all aspects of the procurement and distribution, plus the capital expenditure/research and development (R&D) necessary to establish vaccine manufacture [28] (Makhoana M, Personal communication about the Biovac PPP, Interview; 2014). Such an arrangement can best be described (within the World Bank typology) as a private ownership/private finance initiative in which the private partner owned a controlling share of the assets and, in principle, secured investment funding from private entities. In practice, the required capital funds were sourced through public entities including the Industrial Development Corporation and the Technology Innovation Agency, although some of these funds were made available in the form of loans and should be treated as private funding.

Before the five agreements, including the Supply Agreement, could be signed by the PPP partners, the Transaction Adviser was required, in accordance with the National Treasury Regulations, to undertake a VfM study and determine whether the partnership would indeed result in a positive public outcome without additional cost or risk. Such a study involved an initial options analysis, followed by a more detailed assessment of affordability and VfM. It was concluded that “*Government will be able to retain and build local vaccine manufacturing capacity, allow the transfer of key vaccine R&D and manufacturing skills to South Africa, and build a sustainable, export oriented industry which at the same time can support local initiatives such as the search for an effective HIV vaccine*” [27]. Furthermore, the analysis confirmed that the partnership would result in a significant transfer of risk to the private sector.

The objective of this research is to establish, with hindsight, whether these assessments were in fact accurate and whether the PPP did generate a positive VfM over the period 2010 to 2016.

## Methods

**Fig. 1:**
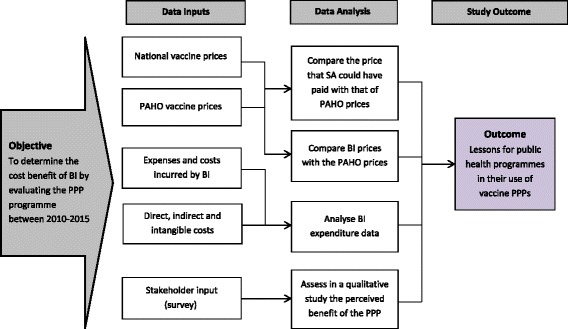
Evaluation approach for this study

The general methodological approach to this study has been based on a concurrent mixed methods approach. In an initial quantitative study, data for prices and product volumes were extracted from secondary data sources (mainly financial statements for the BI) and used to calculate the economic cost of the BI-PPP. Simultaneously, a qualitative study with a purposive sampling strategy was undertaken. A number of key stakeholders in the PPP were identified and then interviewed using a semi-structured questionnaire about their perceptions of the value of the PPP (see below for further details). Similar approaches to the evaluation of public health interventions have become more widely used and are regularly reported in the literature [2, 30]. Each component of the study is now described in more detail. A general overview of the research method is shown in Fig. 1.

### Economic evaluation of immunisation programmes

Specific approaches to the evaluation of vaccination programmes have been covered in the publication ‘Guidelines to Economic Evaluations for Immunisation Programmes’ [31]. The publication covers the processes for economic evaluation with the aim being to present clear, concise, practical and high-quality guidance for performing and presenting the results of economic evaluations [31]. Four types of economic evaluation techniques are recommended, namely cost-minimisation analysis, cost-effectiveness analysis, cost-utility analysis and cost-benefit analysis [32]. Almost all the evaluation techniques estimate costs in a similar style, but measure outcomes or consequences differently [32]. The different ways of measuring benefit lead to a trade-off between the potential impact of the study and its practicality. Although cost minimisation assessments are relatively easy to undertake, they have little benefit in the strategic management of public health. Similarly, long-term social benefits are difficult to assess but have the greatest importance in terms of influence on public health debates.

Key to the rigorous evaluation of a public health intervention is the initial specification of the relevant or even crucial performance indicators [33] and PPPs are no different in this regard. Garvin et al. [34] defined the levels of performance measures derived by the public sector and imposed contractually as broad classifications of desired outcomes required of the private sector. These measures should relate directly to the objectives of the programme.

For the purposes of this discussion, the objectives as listed in Table 1, have been used as the basis for the performance evaluation. However, the list in the table has two obvious omissions, namely timelines and costs. As a result, this study has chosen to benchmark the prices paid by the NDoH against those prices negotiated and published by the Pan American Health Organization (PAHO) as a means of assessing the cost aspects of the economic evaluation. Although the prices are not strictly comparable, since there are differences in terms of whether the goods are landed/not landed, warehoused/not warehoused and other considerations, the PAHO prices provide a consistent basis for the comparison of South African versus international prices. Using this basis, the main research questions of the study now became:

- What were the PAHO and South African prices for vaccines over the reference period?
- What was the price premium paid by the NDoH?
- How were these funds allocated and spent by BI?
- What was the VfM for the public sector as a consequence of the partnership?

The study used the univariate and bivariate methods to graphically display and compare results. International prices were converted to South African Rand values using the average exchange rates in any particular year, adjusted for purchasing power parity, thereby ensuring validity of the comparison. All values in South African Rand were expressed in the form of 2010 values or were converted directly to US$ using the normalisation factors or exchange rate values as shown in Table 2.

**Table 2:** Values for exchange rates and normalisation factors (2010)

Year	2010	2011	2012	2013	2014
Currency conversion (South Africa Rand to US$)	7.638	7.562	8.553	10.037	11.286
Normalisation factor (conversation to 2010 Rand)	1.000	0.938	0.889	0.838	0.793

### Qualitative study

Qualitative studies are useful in business research because they provide clarity on key issues generally not accessible through quantitative data [35]. In this work, a qualitative phase was considered to be important as a means of establishing the phenomenological aspects of the PPP, especially to its key stakeholders such as NDoH and National Treasury. In this respect, qualitative data can provide a more informed and detailed understanding of initiatives such as BI-PPP, thereby generating new ideas and suggestions for improvements [36].

The qualitative component of this study was designed to address the final research question on perceived VfM. A purposive sampling strategy was followed through which key stakeholders in the BI-PPP were identified, including representatives from BI, National Treasury, NDoH, provincial departments of health, the Technology Innovation Agency, the Industrial Development Corporation, the Department of Science and Technology and the Department of Trade and Industry. Once potential respondents had been listed (nine altogether), an interview request was sent through via email, together with a prior informed consent form and a provisional set of questions. The latter were prepared in the form of a semi-structured questionnaire in order to re-assure the respondents that the study was bona fide, but also allow for more general elaboration and discussion where necessary or required [37].

Only five of the sample (55%) agreed to proceed with the interview. It is noted that different modes of interviews were used, including a Skype video calls, face-to-face interviews and telephone calls. In all cases, the conversations were recorded and transcribed. Content analysis was undertaken using ATLAS-ti. In the first case, common themes between the respondents were established and then the transcribed text was annotated according to each theme. This approach facilitated an understanding of the responses and the development of the discursive content in the analysis.

## Results

BI is located in Cape Town, South Africa. The institute has a range of facilities including the Cold Room Warehouse, Quality Control Room, Production, and R&D Pilot Plant. As of the end of 2015, it employed 162 employees and distributed about 11.5 million vaccine vials, equivalent to 46 million doses, per year through its Johannesburg and Cape Town distribution centres.

**Fig. 2:**
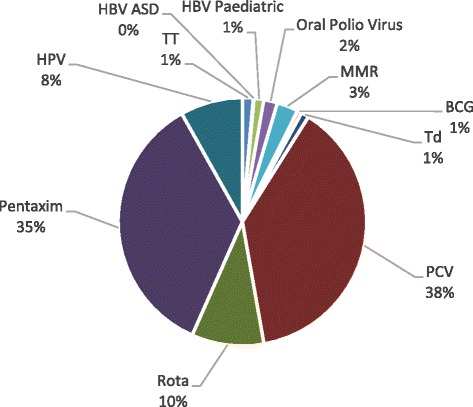
Vaccine components by revenue contribution

The institute procures a broad range of vaccines on behalf of the NDoH, as has already been noted. As of the end of 2014, the largest value vaccines were *Pneumococcal* conjugate vaccine (PCV; 38%), followed by Pentaxim (35%) and finally the rotavirus vaccine (Rota; 10%) (Fig. 2).

**Fig. 3:**
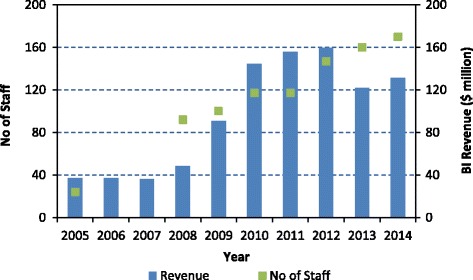
Growth of the Biovac Institute (2005 to 2015)

It is apparent that BI has become a sizeable organisation. In 2004, at the time of its initial establishment, the annual expenditure on public sector vaccines was US$37.5 million [38]. By 2012, this value had grown to US$160 million, an increase of over 400% in real terms, primarily as a consequence of the inclusion of several additional vaccines to the EPI (Fig. 3). Since 2012, the total revenues have declined in dollar terms following the rapid depreciation of the South African Rand relative to the dollar.

The initial projections of the Transaction Adviser did not come close to predicting such an increase in revenue and has changed the initial context for the partnership. Moreover, the required level of human resources and capital investment were similarly under-estimated, with deep implications for the ability of the BI-PPP to deliver on its objectives. The ability of the PPP to accommodate such changes in scope and initial errors of judgement are discussed in the later sections of this article. The finding that the partnership between two such disparate parties is challenging under the best of circumstances, but even more complex within a high-technology and rapidly changing environment, is perhaps the most profound outcome of this PPP project.

### Vaccine purchase prices

The cost-benefit analysis for BI-PPP has been conducted at two levels; firstly, the ability of the institute to access globally competitive prices has been evaluated by the comparison of the BI cost prices against the PAHO prices. Secondly, the monetary value of the premium has been assessed relative to the institute’s contribution to the vaccine supply chain and its progress on the PPP objectives.

Table 3 lists the vaccine price differences per vial (PAHO vs. BI). The BI prices exclude the premiums charged by BI to the NDoH as part of the PPP agreements. There are five important qualifications to this comparison, namely that the tetanus toxoid vaccine is not included in PAHO and was therefore eliminated from the comparison; oral poliovirus was never ordered during the evaluation and therefore also eliminated from the comparison; PAHO does not procure the single-valent vaccines against measles (as is the case in South Africa), so the comparison has been made against the vaccine covering measles, mumps and rubella; similarly, PAHO does not procure an equivalent product to Pentaxim and hence the price for a pentavalent with acellular pertussis has been used in the comparison; and, finally, human papillomavirus was only ordered in 2014 through the evaluation period and hence only included for this year.

**Table 3:** Difference between South African and PAHO vaccine purchase prices (US$ per vial)

Vaccine type	Doses	2010	2011	2012	2013	2014
TT	10	N/A	N/A	N/A	N/A	N/A
HBV Adults	1	0.75	1.31	1.52	1.48	0.49
HBV Paediatric	10	1.84	3.14	3.08	2.75	0.39
OPV	20	0.03	0.71	2.08	1.93	0.72
Measles/MMR	10	3.74	5.12	5.71	5.88	4.28
BCG	20	−0.22	0.50	0.60	0.79	−0.22
Td	10	9.97	10.83	10.12	8.86	5.11
PCV	1	5.09	10.31	5.55	2.74	−0.27
Rota	1	−0.32	0.88	1.49	1.69	−0.61
Pentavalent	1	4.80	8.39	10.28	4.44	−0.43
HPV	1	−20.30	−1.11	−0.87	−1.11	−0.27

*BCG* Bacillus Calmette–Guérin, *HBV* hepatitis B, *HPV* human papillomavirus, *MMR* measles, mumps and rubella, *OPV* oral poliovirus, *PCV Pneumococcal* conjugate vaccine, *Rota* rotavirus vaccine, *Td* tetanus diphtheria, *TT* tetanus toxoid

It is also noted that the vaccines covering tetanus toxoid, hepatitis B for adults and human papillomavirus are not part of the EPI schedule, although the institute does procure and distribute these vaccines on behalf of the NDoH. Furthermore, such direct cost comparisons, as undertaken in this study, can be misleading due to the variations in the components included in the base price. For instance, the standard component is the manufacturer’s ex-factory price but the supplied cost may or may not include freight costs, import tariffs, port fees, customs clearance fees, taxes, mark-ups collected by brokers, distribution costs, overhead costs and procurement costs. The estimated price components can add 30–45% to the original cost from the time the vaccines get dispensed [39]. Furthermore, it is important but difficult to attach a common standard on quality and reliability, including issues such as stock-outs, delivery of damaged goods, safety of vaccines and reliability. Greater care in the delivery chain inevitably adds cost, and a direct comparison of vaccine prices is less meaningful unless the quality criteria are applied consistently.

**Fig. 4:**
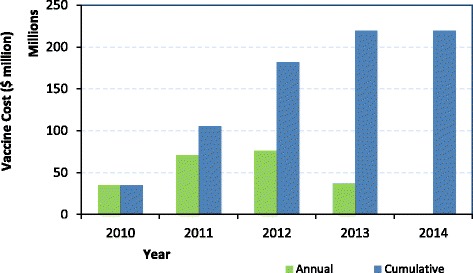
Annual and cumulative difference between PAHO and Biovac Institute total cost for vaccines procured

Based on the values for the number of doses procured annually on behalf of the NDoH by BI, the total value of the additional expense per vaccine which has been incurred as a consequence of independent procurement can be calculated (Table 4). Over the period 2010 to 2014, the BI-negotiated prices were, on average, approximately US$50 million higher for the total portfolio versus PAHO. However, 85% of the price difference can be found in the two newly introduced products of the pentavalent (Pentaxim) and PCV, and in the early years of the study immediately following their introduction. By 2014, the differences on these products were almost negligible relative to the PAHO price, and the two portfolios reached equivalence (Table 4 and Fig. 4).

**Table 4:** Total additional expense of the Biovac Institute via PAHO excluding premium (US$ million)

Vaccine Type	2010	2011	2012	2013	2014	Total
TT	N/A	N/A	N/A	N/A	N/A	N/A
HBV Adult	0.128	0.164	0.171	0.161	0.057	0.679
HBV Paediatric	0.654	1.118	1.111	0.957	0.190	4.030
OPV	0.009	0.357	1.185	0.775	0.448	2.774
Measles/MMR	1.011	1.940	2.996	2.654	2.911	11.513
BCG	−0.052	0.148	0.208	0.464	−0.071	0.697
Td	1.180	1.711	1.756	1.911	0.903	7.461
PCV	14.460	31.290	24.565	8.755	−0.886	78.184
Rota	−0.611	1.917	3.157	3.687	−1.344	6.805
Pentavalent	18.164	32.010	41.175	18.026	−1.678	107.697
HPV	0.000	0.000	0.000	0.000	−0.244	−0.244
Total	34.94	70.66	76.32	37.39	0.29	219.60

*BCG* Bacillus Calmette–Guérin, *HBV* hepatitis B, *HPV* human papillomavirus, *MMR* measles, mumps and rubella, *OPV* oral poliovirus, *PCV Pneumococcal* conjugate vaccine, *Rota* rotavirus vaccine, *Td* tetanus diphtheria, *TT* tetanus toxoid

Based on this trend, it can be argued that BI has been successful in containing the cost of procurement for the EPI vaccines, and that this competence has been strengthened over the period of this study. In other words, procurement prices have not been inflated, either as a means of securing higher levels of funding from the NDoH, given that this funding is charged as a percentage of the procurement value, or as a consequence of a weak bargaining position.

### Value addition services and premium

The second aspect of the cost-benefit ratio is now considered (VfM arising from BI’s contribution to the overall vaccine value chain). The analysis initially confirmed the quantity of the premium charged to the NDoH as permitted within the PPP Supply Agreement. This premium is negotiated on a product-specific basis by BI and varies from 10% to 20%. It is intended to cover all aspects from manufacture (if applicable) to distribution of the packaged/labelled product to the health depots, as may be applicable to the individual products.

**Fig. 5:**
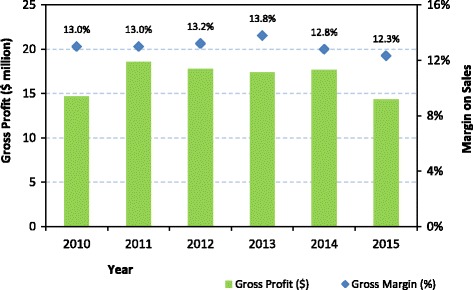
Gross profit and margin over the evaluation period

Figure 5 illustrates the gross margin on sales, essentially equivalent to the premium, received by the institute between 2010 and 2015. The margin averaged at approximately 13%, corresponding to a total value of US$85.7 million over the period of the evaluation or about US$17million per year. It is noted that there are small differences, mostly the consequence of exchange rate fluctuations, between the total margin, as reported in the BI’s Annual Financial Statements, and the total premiums, as calculated from the reported volume of sales and the agreed premium for each product.

**Fig. 6:**
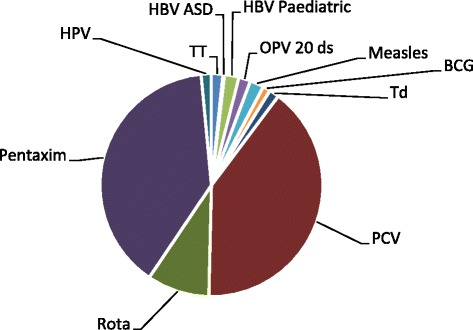
Contribution to the total margin per vaccine product

It has also been possible to calculate the proportion of the overall margin per product (Fig. 6); from the data, it can be deduced that the greatest contributions arise from the largest volume products, namely PCV and the pentavalent.

The critical question is now whether these values represent a fair deal to both parties in the PPP. In addressing this issue, it is important to consider the actual value addition for each product and the typical cost that such a contribution will attract in the overall calculation of the final cost, in this case the cost to the NDoH. Table 5 indicates the premium that was charged and the required activities that took place on the vaccine before it could be distributed. Some vaccines arrived as finished products and were ready for distribution after minimal quality assurance, whilst others arrived incomplete and required filling/packaging.

**Table 5:** Premiums charged and activity per vaccine

Vaccine type	Activity	Agreed premium	Calculated premium
TT	Cold chain distribution	15%	14%
HBV (Adult and Paediatric)	Packaging, labelling and distribution	20%	17%
OPV	Cold chain distribution	15%	14%
Measles	Cold chain distribution	15%	14%
BCG	Packaging, labelling and distribution	20%	17%
Td	Cold chain distribution	15%	14%
PCV	Packaging, labelling and distribution	10%	17%
Rota	Cold chain distribution	10%	14%
Pentaxim	Distribution	15%	12%
HPV	Distribution	15%	12%

*BCG* Bacillus Calmette–Guérin, *HBV* hepatitis B, *HPV* human papillomavirus, *OPV* oral poliovirus, *PCV Pneumococcal* conjugate vaccine, *Rota* rotavirus vaccine, *Td* tetanus diphtheria, *TT* tetanus toxoid

Actual values for costs as a function of value addition are not available, and will generally vary widely within the industry based on geographic location and type/scale of product. In this study, a set of rough guidelines have been used (Table 6). The calculated premiums (Table 5) are based on the required activities and the total volumes, where the latter are important given that there are significant economies of scale. On the basis of these values, it can be concluded that there is good agreement between the actual costs and BI’s value addition to the product. Indeed, if the calculated values are inserted into the calculation for the total premium paid over the reference period, the comparable value amounts to US$106.1 million versus US$85.7 million for the actual value.

**Table 6:** Guidelines for contribution to vaccine price per activity

Activity	Contribution to price (percentage of total price)
Packaging and labelling	5%
Procurement	1%
Contract management	1%
Financial management	2%
Cold chain warehousing	5%
Warehousing	3%
Logistics (distribution)	5%

### Capital investment to develop local manufacture

**Fig. 7:**
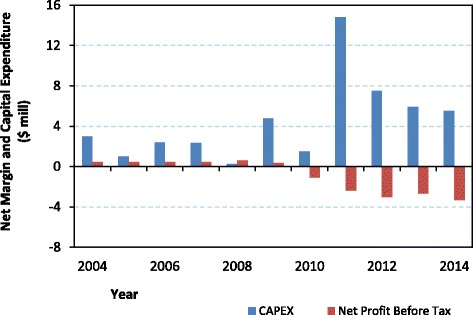
Biovac Institute capital investment and net profit (US$ million; 2004 to 2014)

In the final part of this quantitative evaluation of the cost-benefit ratio for the BI-PPP, we consider the value and focus of its capital investment programme. Over the period 2004 to 2014, a total of US$49 million was invested in the facilities of the institute (Fig. 7).

The main items of this programme are shown in Table 7. The new facilities included an expanded cold room, administration block with quality control laboratories and a clean room facility equipped with state-of-the-art filling equipment, the latter being installed specifically to secure a manufacturing and distribution agreement with Pfizer for the rotavirus vaccine. The funds were obtained from a variety of sources, including the Biovac Consortium (the private partners in the PPP), the Industrial Development Corporation, the Technology Innovation Agency and the Istituto Superiore di Sanità, and covered a range of mechanisms including grants, shareholder loans and general loans. These funds allowed the BI to upgrade its manufacturing and distribution infrastructure, which, as has already been noted, had been badly neglected under the NDoH.

**Table 7:** Main Biovac Institute (BI) capital expenditure items since public–private partnership (PPP) commencement in 2004

Year	Description	Costs (US$ million)	Contributed by
2003	Commencement costs for the PPP	2.99	Biovac Consortium (shareholders loan)
2004	Expansion of the cold room and warehouse (from 849 m^2^ to 1500 m^2^)	1.00	Biovac Consortium (shareholders loan)
2006	Quality control building and laboratories	2.39	Technology Innovation Agency (grant)
2008	Broad funding for BI to progress towards its target of becoming a vaccine manufacturer by 2013	2.56	Technology Innovation Agency (grant)
2009	Warehouse building extension and new administration block	4.76	Industrial Development Corporation (loan)
2011	Construction of clean room	14.82	Istituto Superiore di Sanità and others (grant)
2013	Equipment for packaging, labelling and facility	5.94	Industrial Development Corporation (loan)
2014	Additional facilities to secure manufacturing contract for Rotavirus	5.52	Biovac Consortium (shareholders loan)
Total		39.99	

It is apparent from Table 7 that no capital was made available by the NDoH, the latter being the public partner for the duration of the evaluation period (2010 to 2014). This aspect reflects the categorisation of this partnership, which was earlier described as a private ownership/private finance initiative. The reluctance of the NDoH to make funds available, or to dilute its shareholding as a consequence of equity investments from other potential partners, was a major stumbling block to the realisation of the PPP’s undertakings. The initial estimates of the necessary capital expenditure to transform the vaccine manufacturing assets had been highly optimistic and it was clear that insufficient provision had been made in the agreements to raise additional capital. This aspect is discussed in more detail later.

The second important observation from Table 7 is that no funding could be raised from retained earnings in BI. Indeed, there have been no retained earnings since 2004, with the BI’s expenses being almost equal to the gross margin or premium. In other words, the net profit of the institute after payment of all expenses, including interest charges, has been only slightly positive, and in more recent years, even negative (Fig. 7). The breakdown of the operational expenses is as follows: salary costs (33%), distribution expenses (31%), consumables (13%), depreciation (11%) and maintenance (9%).

This aspect of the finances is disappointing for the PPP since it had been hoped that the premium on sales, as paid by the NDoH, would have been sufficient to cover both the operational expenses and allow for some retained earnings to finance the capital expenditure. The reality was that this expectation was not realised, and the institute was forced to seek commercial loans and grants in order to fund its capital programme.

### Qualitative assessment of the BI by the interviewees

As noted in the methodology section, a number of interviews were held with key stakeholders in order to obtain qualitative feedback on BI’s performance over the evaluation period, and more generally its contribution to vaccine manufacture and supply within South Africa. The results of these discussions are now presented, covering both the respondents’ perceptions of the cost-benefit ratio, and progress in respect of establishing manufacturing infrastructure and a R&D portfolio.

#### Value-for-money

There was a common perception that the PPP could be described as a success because there were no vaccine shortages around the country and supply security had been assured. Most respondents indicated that the PPP delivered VfM to the public of South Africa. Furthermore, it was noted that there had been no interruption in the supply of vaccines to any location in the country.

*“…the vaccine was distributed on a budget, on time and under appropriate conditions, which hasn’t been happening before 2003. Vaccine distribution before the PPP was not reliably sustainable.”*

*“…the distribution is sufficient [*satisfactory*] and works well. Also look at the availability of vaccines in South Africa, there have been one or two hiccups [*shortages or challenges*] [*only*] … we [*the country*] need to have [*vaccine*] supply security…”*

The importance of a private sector partner in this respect was noted.

*“…if government is unable to distribute no matter what its attempts are… and, the private sector does distribute, that is value-for-money. However, in this particular case government could not do what we ended up paying the private sector to do. So there’s no question about value-for-money. The government could not do it* [supply and manufacturing]*. Only the private party could do it…”*

*“…Being a one stop shop to some extent I think has provided value because we are dealing with about eight different suppliers, whereas the Department of Health and just government, in general, has to deal with only one supplier being Biovac.”*

The value of the supply reliability was considered to be ‘beyond estimation’, given that, even if the overall price was higher than it could have been with in-house procurement and distribution, the fact that no child had died as a consequence of not receiving an EPI vaccine was an invaluable contribution.

However, not all participants agreed to the statement on VfM, with one respondent noting that:

*“…South Africa pays too much for vaccines and… it is a victim of the fact that you have only a couple of suppliers”*

This view has been previously stated in the literature [40], and was the main reason that the study considered the comparison between PAHO and BI prices. As noted earlier, although this comment was a valid criticism of the early years covering the introduction of the new vaccines (rotavirus and PCV), by 2014, the local prices in South Africa were highly competitive.

#### Investment in capital equipment and research

During the interviews, the respondents were informed that between 2010 and 2014, BI had received almost US$86 million from the premium charged to the NDoH according to the PPP agreements, and that these funds were being used to support the establishment of local vaccine manufacture, including the construction of the required infrastructure (clean rooms, filling lines, etc.) and a R&D pipeline. The respondents were questioned on their perception of the institute’s progress towards the achievement of the Strategic Equity Partner undertakings, as outlined earlier. In general, the respondents expressed frustration at the slow progress, noting that, as of the beginning of 2015, the PPP had entered its 12th year and that local manufacturing (other than packaging and labelling) was still to take place. BI explained its strategy, and hence the slow progress, as follows:

*“They [*funds received*] were applied for establishing the capability to help pay for the facilities, [*and*] to develop the staff. This is all in preparation for the [*future*] benefits.”*

*“…packaging is maybe the smaller contribution but we are going to filling, and we are going to formulation slowly …”*

*“Currently, almost all vaccines were formulated outside the country. The only formulation currently taking place is only for test and training purposes.”*

*“BI currently have four signed technology transfer agreements. The Pfizer agreement was supposed to be concluded by October 2015. Once all these deals and agreements are secured, Biovac will have had seven EPI vaccines covered.”*

In other words, BI has pursued a phased strategy with backwards integration from the least technology intensive steps. However, it appears that the stakeholders have little understanding of this strategy or the progress that has been made, including the development of the packaging and labelling facility. It appears that BI’s initial strategy had been to invest in human resource development as a way of preparing the institute for its future manufacturing activities. According to a report commissioned by the Biovac Consortium, about 3720 hours were spent on internal training at the BI facility in 2015 and in the same year a total of R2.97 million was spent on external training, which much of this training being based on technical skills development (Frost & Sullivan: Socio-Economic Impact Analysis of the Biovac Institute, unpublished report, 2016).

Similarly, many of the respondents indicated that they were unaware of any benefits from the R&D efforts or even the details of the programme. This view indicates poor communication between the institute and its stakeholders given that there had been some significant achievements. For instance, BI has developed technology for a conjugate vaccine that had recently been licensed to two international companies. The latter companies have successfully commercialised the antigen and achieved WHO prequalification status for their pentavalent vaccine. BI is currently receiving income through licensing fees for the technology. This achievement, which is considered to be a milestone for the institute in its efforts to become an international vaccine company, has not been well publicised.

## Discussion

Globally, the issue of PPPs remains a highly controversial topic [5, 10, 14]. For instance, in the United Kingdom, the liquidation of the construction firm Carillion, at a time when it held 450 contracts with the public sector, highlighted the problems of private outsourcing partners, reigniting public anger towards PPPs (known in the United Kingdom as public finance initiatives) and prompting new calls for their cessation [41]. This controversy persists despite many studies on the main features, benefits and disadvantages of these partnerships [10, 13, 14], with some of the benefits being access to private capital, increased VfM as a consequence of leveraging private sector expertise and efficiency, transfer of risk from the public to the private partner, introducing the concept of social justice and responsibility to private firms, and improved public service delivery as a consequence of public servants being able to focus on their core functions.

On the other hand, the concerns and disadvantages include high transaction and establishment costs, restricted competitive behaviour, higher capital costs, difficulties with relationship management, private sector rent-seeking, poor risk allocation, lower VfM and stifling of innovation [10, 13]. This contradiction in outcomes has resulted in calls for further research to explore the evidence gaps, and particularly to answer the core questions of how to create a successful alliance between public and private actors, how to design and measure the impact of appropriate incentive mechanisms, how to improve inter-actor and inter-project learning, and finally, how to structure governance mechanisms that can combine the best of contractual and relational frameworks [13]. It has been recommended that such questions could be answered through additional longitudinal research [22] and it is within this context that our study of BI has been pursued. In addition, the study has sought to address the question of how PPPs and public procurement could be used as a means of achieving technology transfer in support of local manufacturing, an area known broadly as localisation.

In South Africa, where the PPP programme has been reduced over the past 5 years [42], the establishment of BI has been highly unique. Firstly, as far as the authors are aware, it is the only private ownership/private finance initiative in the country. Secondly, it is effectively a demand-side instrument to build manufacturing capacity in a critical area for the public health sector. The uniqueness of the demand-side nature of the PPP has to be appreciated within the country’s overall policy context. Following the trade negotiations in the 1990s, the country adopted an economic policy framework that opened its borders to international trade and competition whilst simultaneously providing more extensive supply-side support for its manufacturing sector [43]. Recent work on the extant policy mix has shown that supply-side instruments account for more than 98% of the total funding, in comparison to 80% for Canada and 60% for India [44]. Indeed, it appears that BI is one of only three demand-side instruments, alongside the Renewable Energy Independent Power Producers Procurement Programme in the energy sector and the Automotive Production and Development Programme in the automobile sector.

The difficulty for demand-side instruments, and particularly the use of public sector procurement to stimulate local manufacturing (generically referred to as ‘localisation’), is the lack of alignment between the goals of the government department which procures the product (in this case, the NDoH) and the Department of Trade and Industry (DTI), which is responsible for industry promotion. Indeed, the NDoH has been openly and frequently critical of the BI-PPP, stating that, if the DTI wants local industry, then it should pay for its incentivisation.

The results of this study confirm such mixed perceptions about the VfM or cost-benefit ratio of the BI-PPP. In the absence of the PPP, the vaccine value chain (from R&D to manufacture to distribution) in South Africa would undoubtedly have disappeared in its entirety. As of 2003, procurement itself was in jeopardy and the BI-PPP has fulfilled an invaluable function in ensuring supply security since then. However, the cost of the initiative has been carried mostly by the NDoH, whose priority is low-cost public health, and particularly the use of affordable vaccines as a means of addressing key challenges in public health. In this sense, BI is considered to be an unnecessary burden on public health expenditure and the NDoH argued that the budgetary responsibility for vaccine localisation should, at the outset, have been allocated to the DTI or the Department of Science and Technology.

This perspective focuses too narrowly on the benefits of the PPP, which included not only skills development, technology transfer and localisation, but also the maintenance of a reliable and efficient supply chain. As already noted, BI has been successful in negotiating competitive prices for the full spectrum of vaccines, especially towards the end of the evaluation period, and ensuring that the public health depots receive the required doses. The value of this contribution was considered by stakeholders in the sector as immeasurable considering the potential disaster arising from any interruption in supply, particularly with regards to the EPI components.

Notwithstanding the debates about the budgetary responsibility, it is still important to consider the cost to treasury as a whole and the benefits to the South African economy. The former has been separated into the additional procurement cost relative to PAHO prices, which amounted to approximately US$220 million over the evaluation period, and the cost of BI’s operations/capital expenditure, which amounted to US$86 million. The latter has been benchmarked against an estimated cost based on the services provided per vaccine and the associated cost of these services. Although actual or specific industry values are not available, it has been shown that the total cost of BI’s services is less than the benchmark value of US$106 million.

However, a major, and ongoing, concern for the institute is its limited progress towards local filling and manufacture of antigens. An important barrier to this achievement has been the nature of the PPP agreements, which have resulted in insufficient funding to finance the required capital expenditure (the premium has been sufficient to cover only the operational costs), whilst also restricting the ability of the private sector partner to raise either loan or equity capital. The latter is an important learning point for PPPs of this type and arises as a consequence of the short-term duration of the main contracts. The supply agreement, for instance, was initially in place for only 5 years, and although it has since been extended for a second period, the insecurity of this contract prevented BI from raising private funding to support the establishment of local manufacturing facilities.

As a consequence, the institute has followed a cautious and stage-wise strategy to its skills development and capital investment programme, beginning with local repackaging/labelling only, and then backwards integrating into filling, formulating and, hopefully, antigen manufacture. This strategy has been necessitated by the financial and human resource constraints, both of which were largely underestimated in 2003 when the PPP was being conceptualised. The degree of deterioration of the NDoH facilities at this time, and the level of effort required to upgrade these sites to world-class centres, was not apparent to the PPP team and has resulted in the initiative falling somewhat short in reaching the Strategic Equity Partner undertakings.

In the strict sense, this outcome reflects a failure of the PPP to reach its stated objectives and, indeed, if it were to be assumed that the governance structure was entirely contractual, the PPP would have been terminated quite soon after the initial 3-year cycle. However, it is apparent that the NDoH recognised the difficulty of the initial targets and allowed a more relational governance structure to evolve within the PPP. As noted earlier, such dynamics are not unusual in public–private procurement arrangements and have been previously reported [22]. The transition to a more flexible governance structure undoubtedly led to some relaxation of the contractual conditions as these were implicitly re-negotiated as a consequence (of the change). BI continued to supply vaccines, and receive a premium on the cost of procurement, despite failing to meet the target regarding local filling and manufacture of antigens.

## Conclusions

Over the period 2010 to 2014, BI successfully procured and distributed vaccines and received an income of US$86 million, equivalent to an average cost premium of 12%, as per the terms of its supply agreement with the NDoH. Moreover, it became increasingly able to supply vaccines to the public health system at globally competitive prices and undertook local R&D, the latter in one case leading to a novel conjugate vaccine that has been licensed to two international companies and for which the institute receives royalty revenue.

The entire premium was used to finance BI’s operational expenses, with insufficient retained earnings with which to build world-class local manufacturing facilities. As a result, the institute was required to raise this capital through loans and grants. Unfortunately, these efforts were hindered by the short-term nature of the supply agreement, which prevented the entry of equity partners or other investors to any significant extent. This aspect of the PPP led BI to adopt a slow and stage-wise investment strategy, beginning with repackaging/labelling and only gradually migrating upstream to more value-adding activities.

In summary, the quantitative and qualitative approaches of this study have concluded that a positive cost-benefit or VfM outcome was achieved by the BI over the evaluation period. Beneficial outcomes include a capability to negotiate internationally competitive prices, an integrated distribution network, uninterrupted vaccine supply, technology transfer, skills development, a successful R&D product and the infrastructure for local manufacture. Although it is beyond the scope of this study to comment on the future management of the PPP, it is concluded that, although more could have been achieved, the results to date indicate that the initiative has acted in the interest of the public, particularly with respect to ensuring VfM from the expenditure of public funds.

Future recommendations for policy-makers and practitioners include a more cautious and incremental approach to the development of a local vaccine value chain, with clearer consideration of the intermediary steps and articulation thereof in a detailed set of contractual documents containing well-defined goals and associated performance incentives. Although an initiative such as BI cannot be compared to the construction of a road or a building, and could not be managed through a contractual governance structure, it is evident from this study that dominance of relational governance in the later stages of the PPP led to slippage in the shareholder obligations. A more balanced mixture could only have been followed if the project had been approached incrementally, and involved a large set of public shareholders in addition to the NDoH.

## Abbreviations

BI: Biovac Institute
DTI: Department of Trade and Industry
EPI: Expanded Program on Immunisation
NDoH: National Department of Health
PAHO: Pan American Health Organization
PCV: *Pneumococcal* conjugate vaccine
PPP: public–private partnership
R&D: research and development
